# Student nurses’ perceptions of their educational environment at a school of nursing in Western Cape province, South Africa: A cross-sectional study

**DOI:** 10.4102/curationis.v42i1.1914

**Published:** 2019-04-08

**Authors:** Katlego D.T. Mthimunye, Felicity M. Daniels

**Affiliations:** 1 School of Nursing, University of the Western Cape, Cape Town, South Africa

## Abstract

**Background:**

Educational environments have been found to bear a substantial relationship with the academic performance and success, as well as the retention, of students.

**Objectives:**

The study objectives were to (1) evaluate the educational environment as perceived by undergraduate nursing students at a school of nursing (SON) in Western Cape province and (2) investigate whether the educational environment, or components thereof, is perceived negatively or positively among undergraduate nursing students of different year level, gender, home language and ethnicity.

**Method:**

A quantitative research method with a cross-sectional design was implemented. Data were collected from 232 undergraduate nursing students from a SON at a university in Western Cape province, South Africa. The subscales and the items of the educational environment questionnaire were compared among undergraduate nursing students. Data were analysed by means of the IBM Statistical Package for Social Sciences (IBM SPSS-24) using analysis of variances (ANOVAs), independent-sample *t*-tests, mean scores, standard deviations and percentages.

**Results:**

The mean score attained for the entire participant group was 195 (standard deviation [SD] = 24.2) out of 268 (equivalent to 72.8% of maximum score), which indicated that the educational environment was perceived substantially more positively than negatively. The overall mean score was significantly higher (*p* < 0.05) for male students (*M* = 202; *SD* = 21) and for black students (*M* = 202; *SD* = 21). The digital resources (DR) subscale was the only subscale with a statement or item that was rated as absolute negative (*M* = 1.9; *SD* = 0.9).

**Conclusion:**

The educational environment at the institution concerned was perceived as predominantly positive by its undergraduate nursing students. Although the educational environment was predominantly perceived as positive, the results of this study also indicated that enhancements are required to improve the physical classroom conditions, skills laboratories, DR and the implemented teaching and learning strategies. It is vital for university management to prioritise the creation of an educational environment which would ensure that quality learning takes place.

**Keywords:**

student’s perceptions; educational environment; nursing education; Western Cape; South Africa.

## Introduction

Teaching and learning in nursing education is undergoing substantial transformation worldwide (Aiken 2011; Benner 2012; World Health Organization 2013). These transformations challenge nursing schools to implement new strategies to facilitate high-quality teaching and learning (Benner 2012). The educational environment in particular plays a central role in the process of teaching and learning (Arzuman, Yusoff & Chit 2010; Cleveland & Fisher 2014; Davies et al. 2013; Korucu & Alkan 2011). The educational environment in nursing comprises both practical and theoretical learning settings (Billings & Halstead 2015). It also incorporates a variety of basic provisions such as the physical infrastructure, the teaching and learning processes, school resources or materials and the teacher–student relationship (Miles, Swift & Leinster 2012). In addition, for nursing students, an ideal educational environment should promote critical thinking and lifelong learning (Billings & Halstead 2015; Davis & Kimble 2011). However, it is also necessary to be aware that ‘demotivating elements such as perceived bias, poor role models, information overload, teacher-centered or disorganized teaching need to be identified and eliminated’ (Veerapen & McAleer 2010:2). As a further cautionary observation, Bruce, Klopper and Mellish (2011) suggest that nursing education requires a modern educational environment which is focused more on the learning paradigm than on the teaching paradigm.

## Background

The literature reveals that educational environments have an impact on students’ levels of success, achievement, contentment and motivation (Arzuman et al. 2010). Furthermore, Till (2005) and Arzuman et al. (2010) have suggested that students’ satisfaction with their educational environment is associated with the depth and quality of learning. Arzuman et al. (2010) and Al Ayed and Sheik (2008) reported that educational environment domains correlate positively with the academic success and, ultimately, the retention of students. Therefore, students’ perceptions of their educational environment serve as a valuable foundation for transforming and improving the quality of the educational environment. In addition, Baeten et al. (2010) and Cheon et al. (2012) reported that students’ perceptions of their learning environment have a significant impact on the learning strategies that they may adopt. For example, Baeten et al. (2010) reported that students in the community and health sciences faculty exhibited a deep learning approach towards their learning. Therefore, an environment that promotes quality and deep learning is vitally significant in ensuring successful teaching of, and learning by, nursing students. Moreover, the improvement of the overall educational environment is likely to have a significant influence on the academic performance and retention of the nursing student (Al Ayed & Sheik 2008; Arzuman et al. 2010; Till 2005).

Evaluation of the students’ perceptions towards their educational environment at the school of nursing (SON) would aid nurse educators and faculty staff in measuring the quality of the teaching and learning taking place (Denz-Penhey & Murdoch 2009). Although numerous studies have been conducted globally, evaluating the perceptions of medical as well as nursing students’ perceptions of their educational environment (Colbert-Getz et al. 2014; Ostapczuk et al. 2012; Rahman et al. 2015; Yusoff 2012), we are not aware of any studies evaluating nursing students’ perceptions of their educational environments either in South Africa (SA) or at the identified university in the Western Cape province.

## Objectives

The objectives of this study were to:

- evaluate the educational environment as perceived by undergraduate nursing students at a SON in the Western Cape province of SA
- investigate whether the educational environment, or components thereof, was perceived negatively or positively among undergraduate nursing students of different year level, gender or ethnicity.

## Methods

### Research design

A quantitative research method with a cross-sectional design, using a researcher-developed survey, was used.

### Context of the study

This study was conducted in the SON at a university in the Western Cape province of SA. The SON offers a range of undergraduate and postgraduate programmes. The undergraduate programmes offered by the SON were the main focus of this study, and these include the 4-year Bachelor of Nursing (BN) and 5-year Bachelor of Nursing Foundation (BNF) programmes.

### Population, sample and sampling technique

#### Inclusion criteria

As a result of the apartheid system and segregation policies in SA, the racial^1^ classification of population groups differs slightly from general classifications used around the world (Cornell & Hartmann 2007). For example, the term ‘coloured’ in a South African context refers to a population group of mixed race (Moultrie & Timæus 2003). In SA, the predominant racial groupings are classified as black, white, coloured and Indian. The study population included all students registered for the BN and BNF programmes at the university concerned.

#### Sampling technique

Stratified random sampling was used to ensure that all levels of the BN and BNF programmes were adequately represented (Table 1). In stratified random sampling,

the population of interest is first divided into two or more groups based on characteristics that are important to the study, and then members within each group are randomly selected. (Macnee & McCabe 2008:128)

**TABLE 1:** Summary of study population, sample and response rate.

Year level	Population (*N*)	Sample (*n*)	Response *n* (%)
First-year Bachelor of Nursing Foundation (BNF 1)	74	19	14 (4.88)
Second-year Bachelor of Nursing Foundation (BNF 2)	45	11	9 (3.14)
First-year Bachelor of Nursing (BN 1)	221	56	49 (17.07)
Second-year Bachelor of Nursing (BN 2)	303	77	62 (21.60)
Third-year Bachelor of Nursing (BN 3)	240	61	48 (16.72)
Fourth-year Bachelor of Nursing (BN 4)	248	63	50 (17.42)

**Total**	**1131**	**287**	**232 (80.84)**

#### Sample size

The sample size equation *n* = (*p*) (1−*p*) (*Z*)^2^/*e* ^2^ with a 95% confidence level (*Z* = 1.96), an error rate (*e*) of 5% and a proportion of the target population (*p* = 50%) revealed that a sample of 384 is required to represent the population (Dean, Sullivan & Soe 2013). An adjusted sample size of 287 was derived using the equation *n_a_* = *n*/(1 + (*n* – 1)/*N*), where the population (*N*) is 1131 (Dean et al. 2013). Furthermore, the equation *c* = (*N*_s_/*N*)*n*_a_ was used to calculate the sample size within each strata, where *c* is the sample size for stratum, *N*_s_ is the population size for stratum, *N* is the total population size and *n*_a_ is the total sample size (Dean et al. 2013). Table 1 summarises the study population sample as well as the response rate.

### Research instrument and data collection

Data were collected using a researcher-developed questionnaire that was administered to the sampled undergraduate (BN and BNF) nursing students. The questionnaire consisted of a total score of 268 for 67 items on a four-point Likert scale (where 1 = strongly disagree, 2 = disagree, 3 = agree and 4 = strongly agree). For interpretation of the overall survey score, the following overall scores were considered based on quadrant parameters: 0–67 = very poor; 68–134 = poor; 135–201 = good; 202–268 = excellent.

The survey consisted of demographic factors and eight subscales that were used to measure nursing students’ perception of the educational environment. The instruments’ subscales and measurements included the following:

- physical classroom environment (PCE) – 11 items; maximum score = 44
- skills laboratory (SL) (on-campus) – six items; maximum score = 24
- SL (off-campus) – six items; maximum score = 24
- university library (UL) – five items; maximum score = 20
- digital resources (DR) – seven items; maximum score = 28
- teaching and learning climate (TLC) – nine items; maximum score = 36
- teaching and learning strategies (TLS) – 11 items; maximum score = 44
- nursing curriculum (NC) – 12 items; maximum score = 48.

In this study, TLC refers to professional relationships among students and educators, whereas TLS refers to the teaching and learning methodologies implemented at the SON.

Items with a mean score of 3.0 or more indicate absolute positive aspects. Items with a mean score of 2.0 or below indicate absolute negative aspects and need immediate intervention. Items with a mean score of between 2.0 and 3.0 are aspects of the educational environment that warrant improvement.

### Reliability of research instrument

A pilot test of the instrument preceded the actual data collection to ensure reliability of the data collection instrument. Perneger et al. (2015) suggested that, to produce significant results from a pretest, a minimum sample size of 30 participants is recommended. Questionnaires were administered to 30 undergraduate nursing students (selected via convenience sampling) who were not included in the main study. The questionnaire was then administered to the same group 2 weeks later to ensure test–retest reliability (Polit & Beck 2010). The test–retest reliability revealed an intraclass correlation coefficient of 0.954, indicating an excellent correlation coefficient (Field 2013). Finally, the reliability process involved calculating the internal consistency reliability which revealed a Cronbach’s alpha coefficient of 0.945. The individual items of the instrument revealed a Cronbach’s alpha coefficient ranging from 0.943 to 0.945. This Cronbach’s alpha coefficient confirms that the items being measured were internally reliable (Field 2013). According to Tavakol and Dennick (2011), a significant Cronbach’s alpha coefficient (≥ 0.70) adds to the validity and accuracy of the instrument. Thus, an instrument cannot be valid unless it is reliable (Tavakol & Dennick 2011).

### Validity of the instruments

The content validity of the questionnaire was established by the research supervisor (an expert in teaching and learning) and a statistician. Their inputs were implemented to improve the items in the questionnaire. In addition, face validity was conducted by 30 undergraduate nursing students during the pilot test of the instrument to ensure accurate interpretation of the content. During the face validity, none of the participants requested verbal assistance and they responded to all the items included in the instrument. In general, the participants in this pilot test reported that the instruction and the content of the instrument were well defined.

### Data processing and analysis

Data were analysed using the IBM Statistical Package for Social Sciences (IBM SPSS-24). Missing values were dealt with by replacing them with the median of nearby points to avoid errors and skewness of the data. Descriptive and inferential statistics were performed by means of frequencies, standard deviations (SDs) and percentages for the total score of the questionnaire and subscale scores of the whole sample as well as the specific BN and BNF groups, ethnic group and gender. For dichotomous variables (home language and gender), comparisons of overall and subscale mean scores were achieved through a series of independent-sample *t*-tests. For variables with more than two values (ethnicity and year level of study), a series of one-way analysis of variances (ANOVAs) were performed to compare all the groups. Where one-way ANOVAs were not possible owing to violation of the significant homogeneity of variances ( *p* < 0.05), the alternative statistical test – Welch ANOVA – was used. The significance level for ANOVA was established at *p* < 0.05. Where significant ( *p* < 0.05) differences between the groups were found, a post hoc Tukey’s honestly significant difference (HSD) test for multiple comparisons was used to verify where the variances occurred between the groups. Where no significant ( *p* > 0.05) differences between the groups were found (equal variances not assumed), a nonparametric Games–Howell post hoc test set for multiple comparisons was used to verify where the variances occurred between the groups.

### Ethical considerations

Ethics in research is a serious matter and researchers need to adhere to strict rules (Denscombe 2014). Participation in the study was voluntary and was based on participants’ consent. Ethics clearance (HS17/1/42) was obtained from the university ethics committee. Permission to conduct the study at the identified SON was obtained from the registrar of the university as well as the director of the SON.

## Results

### Participants

A total of 232 (80.84%) students out of the 287 stratified random sample completed the survey. The demographic data revealed that of the 232 students, 182 (78.45%) were females and 50 (21.55%) were males. More than half (*n* = 132; 56.90%) of the participants were of black ethnicity, followed by coloured students (*n* = 74; 31.90%), white students (*n* = 18; 7.76%) and Indian students (*n* = 4; 1.72%). The category classified as ‘other’, which included all the students who did not belong to any of the four main categories as classified by Statistics South Africa (2016), comprised four (1.72%) students. The youngest participant was 18 years old and the oldest was 49 years old. The mean age of the study participants was 23.02 (*SD* = 5.11) years. Of the 232 participants, 63 (27.16%) spoke English as their home language, whereas the remaining 169 (72.84%) spoke other languages including IsiXhosa and Afrikaans.

### Overall mean scores by year level of study

A one-way between-groups ANOVA was performed to compare the nursing students’ perceptions regarding their educational environment for each year level of study in the undergraduate programme. Subscale means and SDs for the whole sample as well as for each year level are summarised in Table 2. For this ANOVA, the outcome variables were found to be normally distributed and equal variances were assumed except for the UL subscale, which revealed a Levene’s statistic of *F*_(5, 226)_ = 4.8; *p* < 0.000. As the assumption of homogeneity of variance was not met for the UL subscale, the Welch statistical test was performed and the results revealed Welch’s *F*_(5, 55.08)_ = 1.81, which was found to be not significant (*p* = 0.127). The Games–Howell post hoc comparison test revealed that there was no statistical difference between all unique pairwise comparisons.

**TABLE 2:** Mean (standard deviation) and overall scores by year level.

Domains	BNF 1	BNF 2	BN 1	BN 2	BN 3	BN 4	All	*F*	*p*	Tukey’s HSD < 0.05
Physical classroom environment (PCE)	32 (3.3)	33 (3.2)	32 (4.8)	31 (5.4)	30 (5.9)	30 (5.9)	31 (5.4)	1.303	0.264	-
Skills laboratory (SL) (on-campus)	18 (2.0)	18 (2.3)	19 (3.2)	16 (3.7)	15 (3.7)	16 (3.2)	17 (3.6)	6.341	0.000*	BN 1–BN 2, BN 1–BN 3, BN 1–BN 4
Skills laboratory (SL) (off-campus)	18 (3.0)	18 (2.5)	18 (3.1)	16 (3.3)	16 (3.8)	16 (3.2)	17 (3.4)	4.242	0.001*	BN 1–BN 2, BN 1–BN 3, BN 1–BN 4
University library (UL)	16 (2.1)	15 (2.3)	16 (2.5)	16 (2.7)	14 (4.0)	16 (2.8)	16 (3.0)	1.81	0.127	-
Digital resources (DR)	20 (3.0)	19 (2.7)	21 (3.6)	19 (3.4)	17 (3.6)	19 (3.7)	19 (3.7)	4.982	0.000*	BN 3–BN 1, BN 3–BN 2
Teaching and learning climate (TLC)	28 (3.7)	27 (3.7)	30 (3.8)	28 (4.7)	26 (5.3)	25 (5.4)	27 (3.7)	7.254	0.000*	BN 1–BN 3, BN 1–BN 4, BN 2–BN 4
Teaching and learning strategies (TLS)	34 (3.3)	44 (4.7)	34 (4.6)	33 (5.6)	31 (5.3)	31 (6.4)	32 (5.5)	2.773	0.019*	BN 1–BN 4
Nursing curriculum (NC)	38 (5.0)	37 (6.5)	39 (4.1)	37 (5.5)	36 (5.4)	33 (6.4)	37 (5.7)	5.469	0.000*	BN 4–BN 1, BN4–BN2

**Total**	**202 (12)**	**201 (22)**	**208 (19)**	**197 (23)**	**186 (25)**	**186 (26)**	**195 (24.2)**	**7.098**	**0.000**	**BN 1–BN 3, BN1–BN4**

***n***	**14**	**9**	**49**	**62**	**48**	**50**	**232**	**-**	**-**	**-**

BNF 1, first-year Bachelor of Nursing Foundation; BNF 2, second-year Bachelor of Nursing Foundation; BN 1, first-year Bachelor of Nursing; BN 2, second-year Bachelor of Nursing; BN 3, third-year Bachelor of Nursing; BN 4, fourth-year Bachelor of Nursing; HSD, honestly significant difference; *F*, variation between group means; *p*, significance;

* , *p* > 0.05.

The total mean score for all the students who participated in the present study was 195 (72.8% of the maximum score), with an SD of 24.2. These results indicate that, generally, the educational environment, as perceived by undergraduate nursing students at the identified university, was good but could be improved upon. The total scores varied significantly between year levels (*F*_(5, 226)_ = 7.098; *p* < 0.000). Post hoc analysis using Tukey’s HSD test indicated that first-year BN students had a significantly positive perception (*p* < 0.000) about their overall educational environment as compared with the senior students (third- and fourth-year BN students).

Skills laboratory (on-campus) scores varied significantly between the year levels of the undergraduate programme (*F*_(5, 226)_ = 6.341; *p* = 0.000). SL (off-campus) scores varied significantly between the year levels (*F*_(5, 226)_ = 4.242; *p* = 0.001). Likewise, taken together, the results of Tukey’s post hoc HSD statistics for both on- and off-campus SL indicated that generally first-year BN students have a significantly positive (*p* < 0.05) perception about the skills laboratories compared with second-, third- and fourth-year BN students.

Digital resources mean scores varied significantly between year levels (*F*_(5, 226)_ = 4.982, *p* = 0.000). Post hoc Tukey’s HSD statistics indicated that first- and second-year BN students had a significantly positive perception (*p* < 0.005) about the DR, as compared with the third-year BN students.

Teaching and learning climate mean scores varied significantly (*F*_(5, 226)_ = 7.254, *p* = 0.000). Post hoc Tukey’s HSD statistics suggest that the first-year BN students had a significantly positive perception (*p* < 0.005) about the TLC as compared with their senior third- and fourth-year BN students. Likewise, Tukey’s post hoc HSD statistics revealed that second-year BN students had a significantly positive perception (*p* < 0.005) about the TLC as compared with fourth-year BN students.

The TLS mean score varied significantly (*F*_(5, 226)_ = 2.773, *p* = 0.019). Tukey’s post hoc HSD statistics results indicated that first-year BN students had a significantly positive perception (*p* = 0.032) towards the TLS implemented at the identified university as compared with the fourth-year BN students.

The mean score of the students’ perceptions regarding the NC varied significantly (*F*_(5, 226)_ = 5.469, *p* = 0.000). Tukey’s post hoc HSD test results indicated that first-year BN students and second-year BN students had a significantly positive perception (*p* < 0.05) regarding the NC at the identified university as compared with the fourth-year BN students.

### Overall mean score by ethnicity

A one-way between-groups ANOVA was performed to compare the nursing students’ perceptions regarding their educational environment for each ethnic group. Participants were divided into five groups based upon their ethnic demographics (black, coloured, Indian, white and other). Subscale means and SDs for ethnicity are displayed in Table 3. For this ANOVA, the outcome variables were found to be normally distributed and equal variances were assumed except for PCE, NC and total score, which revealed the following Levene’s statistics respectively: *F*_(4, 227)_ = 2.59, *p* = 0.038; *F*_(4, 227)_ = 3.36, *p* = 0.011 and *F*_(4, 227)_ = 4.25, *p* = 0.002. As the assumption of homogeneity of variance was not met for PCE, NC and overall score, Welch statistics were performed. The results for PCE revealed Welch statistic *F*_(4, 11.15)_ = 2.38, which was found to be not statistically significant (*p* = 0.114). The Games–Howell post hoc comparison test revealed that there was no statistical difference between all ethnic groups in pairwise comparisons. The results for NC revealed Welch statistic *F*_(4, 11.11)_ = 4.93, which was found to be statistically significant (*p* = 0.016). Post hoc comparison Games–Howell statistics indicated that black students had a more positive perception (*p* < 0.05) towards the NC at the university compared with their coloured counterparts. Welch statistic results for the overall score by ethnicity (*F*_(4, 11.09)_ = 5.25) were found to be significant (*p* = 0.013). The post hoc comparison Games–Howell statistic indicated that, overall, black students had a more positive perception (*p* < 0.05) towards their educational environment compared with their coloured counterparts.

**TABLE 3:** Mean (standard deviation) and overall score by ethnicity.

Domains	Black	Coloured	Indian	White	Other	*F*	*p*	Tukey’s HSD < 0.05
Physical classroom environment (PCE)	32 (5)	30 (5)	24 (7)	30 (6)	24 (10)	2.382	0.114	-
Skills laboratory (SL) (on-campus)	18 (3)	16 (4)	16 (6)	16 (2)	15 (5)	4.847	0.001*	Black–coloured
Skills laboratory (SL) (off-campus)	18 (3)	16 (3)	15 (6)	17 (2)	15 (6)	3.208	0.014*	Black–coloured
University library (UL)	16 (3)	16 (3)	17 (2)	15 (2)	14 (3)	0.361	0.836	-
Digital resources (DR)	19 (4)	19 (3)	15 (6)	19 (4)	16 (4)	2.827	0.026*	-
Teaching and learning climate (TLC)	28 (5)	26 (5)	26 (6)	27 (6)	20 (6)	5.809	0.000*	Black–coloured, black–other, white–other
Teaching and learning strategies (TLS)	33 (5)	31 (5)	27 (6)	30 (6)	25 (7)	6.235	0.000*	Black–coloured, black–other
Nursing curriculum (NC)	38 (5)	35 (5)	30 (11)	37 (7)	28 (8)	4.927	0.016*	Black–coloured

**Overall**	**202 (21)**	**188 (22)**	**169 (45)**	**190 (26)**	**156 (46)**	**5.249**	**0.013**	**Black–coloured**

***n***	**132**	**74**	**4**	**18**	**4**	**-**	**-**	**-**

* , *p* > 0.05; HSD, honestly significant difference; *F*, variation between group means; *p*, significance.

The mean score of the students’ perceptions regarding the on-campus (*F*_(4, 227)_ = 4.85, *p* = 0.001) and off-campus (*F*_(4, 227)_ = 3.21, *p* = 0.014) skills laboratories by ethnicity varied significantly. The post hoc Tukey’s HSD test results indicate that black students had a positive perception (*p* < 0.05) regarding both on-campus and off-campus skills laboratories compared with their coloured counterparts.

The mean score of the students’ perceptions regarding DR by ethnicity varied significantly (*F*_(4, 227)_ = 2.83, *p* = 0.026). Tukey’s post hoc HSD test results revealed that there were no significant statistical differences between all unique pairwise comparisons.

The mean score of students’ perceptions of the TLC by ethnicity varied significantly (*F*_(4, 227)_ = 5.81, *p* = 0.000). Tukey’s post hoc HSD statistics indicated that black students had a more positive perception (*p* < 0.05) regarding the TLC at the identified university compared with their coloured counterparts as well as the category classified as ‘other’. Tukey’s post hoc HSD results also revealed that white students had a more positive perception (*p* < 0.05) regarding the TLC compared with students classified in the category of ‘other’.

The mean score of students’ perceptions regarding the TLS implemented at the identified university varied significantly (*F*_(4, 227)_ = 6.24, *p* = 0.000). Tukey’s post hoc HSD statistics indicated that black students had a significantly positive perception (*p* < 0.05) of the TLS compared with coloured students and those classified as ‘other’.

### Gender differences

An independent-samples *t*-test was performed to compare the nursing students’ perceptions regarding their educational environment among male and female undergraduate nursing students. The overall mean score was significantly (*p* < 0.05) higher for male students than for female students (*t*(230) = 2.3, *p* = 0.022). These results indicated that male students’ perceptions regarding their teaching and educational environment were more positive compared with their female counterparts. A summary of the independent-sample *t*-test for comparison of the subscale scores and gender is presented in Table 4.

**TABLE 4:** Mean score (standard deviation) and overall scores by gender (*N* = 232).

Domains	Female	Male	*t*	*p*
Physical classroom environment (PCE)	31 (5)	32 (5)	2.089	0.038*
Skills laboratory (SL) (on-campus)	16 (4)	18 (3)	2.100	0.037*
Skills laboratory (SL) (off-campus)	17 (3)	18 (3)	1.789	0.075
University library (UL)	16 (3)	16 (3)	0.481	0.631
Digital resources (DR)	19 (4)	19 (4)	0.506	0.614
Teaching and learning climate (TLC)	27 (5)	28 (4)	1.390	0.166
Teaching and learning strategies (TLS)	32 (6)	33 (5)	1.624	0.106
Nursing curriculum (NC)	36 (6)	38 (5)	1.994	0.047*

**Overall**	**193 (25)**	**202 (21)**	**2.302**	**0.022**

*n*	**183**	**50**	**-**	**-**

* , *p* > 0.05; *t*, Gosset’s Student distribution (difference between population means); *p*, significance.

### Overall mean scores for undergraduate students

The mean scores for UL were the highest (3.1 out of 4), followed by TLC and NC (3.0 out of 4 for both subscales). The remaining five subscales (PCE, SL [on-campus], SL [off-scampus], DR and TLS) revealed mean scores below 3 out of 4. The results revealed that the weakest subscale was DR with a mean score of 2.7 out of 4. Furthermore, the DR subscale was the only subscale with a statement or item that was rated an absolute negative. Table 5 summarises the mean scores and interpretation of items under investigation.

**TABLE 5:** Mean score (out of 4) of the items under study domains.

Items	Mean (SD)	Interpretation
**Physical classroom environment**		
(1) Classrooms are pleasant places to work	2.8 (0.8)	Could be improved
(2) Lighting is adequate and there is no glare	3.0 (0.8)	Absolute positive
(3) Ventilation is sufficient and the temperature is appropriate	2.7 (0.9)	Could be improved
(4) There is adequate space for movement	3.1 (0.8)	Absolute positive
(5) Furniture is arranged to best effect for different activities	2.6 (0.8)	Could be improved
(6) Equipment and materials are easily accessible (computer, lighting system, projector, overhead projector)	2.6 (0.9)	Could be improved
(7) Adequate seating arrangements for students	2.9 (0.8)	Could be improved
(8) Students have adequate personal workspace	2.9 (0.7)	Could be improved
(9) Students can easily see the teacher and the black or white board	3.1 (0.7)	Absolute positive
(10) Furniture is suitable and well maintained	2.4 (0.8)	Could be improved
(11) Sound level in classroom is conducive or favourable to learning	2.8 (0.8)	Could be improved
**Mean score**	**2.8 (0.5)**	**Could be improved**
**Skills laboratory: on-campus**		
(12) Adequate in size	2.6 (0.9)	Could be improved
(13) Adequate lighting	3.2 (0.6)	Absolute positive
(14) Adequate ventilation	2.8 (0.8)	Could be improved
(15) Equipped with appropriate and sufficient equipment necessary for students’ practice of required clinical skills	2.8 (0.8)	Could be improved
(16) Equipped with appropriate and sufficient supplies (stock) necessary for student’s practice of required clinical skills	2.8 (0.8)	Could be improved
(17) Accessible to students outside regularly scheduled class times	2.6 (0.9)	Could be improved
**Mean score**	**2.8 (0.6)**	**Could be improved**
**Skills laboratory: off-campus**		
(18) Adequate in size	2.8 (0.8)	Could be improved
(19) Adequate lighting	3.1 (0.7)	Absolute positive
(20) Adequate ventilation	2.9 (0.8)	Could be improved
(21) Equipped with appropriate and sufficient equipment necessary for student’s practice of required clinical skills	2.9 (0.7)	Could be improved
(22) Equipped with appropriate and sufficient supplies (stock) necessary for student’s practice of required clinical skills	2.8 (0.7)	Could be improved
(23) Accessible to students outside regularly scheduled class times	2.2 (0.9)	Could be improved
**Mean score**	**2.8 (0.6)**	**Could be improved**
**University library**		
(24) Institutional library personnel offer orientation and demonstration of the library services	3.2 (0.8)	Absolute positive
(25) Library personnel provide assistance to students when needed	3.1 (0.8)	Absolute positive
(26) Library is user friendly for nursing students	3.0 (0.8)	Absolute positive
(27) Library has sufficient materials to support programme or classroom assignments	3.1 (0.8)	Absolute positive
(28) Library operating hours are convenient for students	3.3 (0.8)	Absolute positive
**Mean score**	**3.1 (0.6)**	**Absolute positive**
**Digital resources**		
(29) Computer laboratories are adequate to support learning (research, assignment completion, etc.)	2.9 (0.8)	Could be improved
(30) Effective use of various mediums such as online teaching and learning (Ikamva)	3.1 (0.7)	Absolute positive
(31) Adequate resources for students during online assessments	2.9 (0.8)	Could be improved
(32) E-learning support services are readily accessible to all students	2.9 (0.7)	Could be improved
(33) Computer laboratories are available outside regular classroom hours	2.8 (0.9)	Could be improved
(34) Off-campus internet connectivity (Wi-Fi) is readily accessible	1.9 (0.9)	Absolute negative
(35) On-campus internet connectivity (Wi-Fi) is readily accessible	2.6 (0.9)	Could be improved
**Mean score**	**2.7 (0.5)**	**Could be improved**
**Teaching and learning climate**		
(36) Lecturers or clinical facilitators are approachable	3.2 (0.7)	Absolute positive
(37) Lecturers or clinical facilitators are concerned with developing my competence	3.2 (0.7)	Absolute positive
(38) Lecturers or clinical facilitators are able to communicate well with students	3.0 (0.8)	Absolute positive
(39) Lecturers or clinical facilitators have shown patience towards students	3.0 (0.8)	Absolute positive
(40) Lecturers or clinical facilitators provide good feedback to students	2.9 (0.8)	Could be improved
(41) Lecturers or clinical facilitators give students constructive criticism	3.0 (0.8)	Absolute positive
(42) Lecturers or clinical facilitators are well prepared for classes	3.2 (0.8)	Absolute positive
(43) I feel free to ask whatever question I want in class	2.9 (0.8)	Could be improved
(44) The environment encourages me to learn	2.9 (0.8)	Could be improved
**Mean score**	**3.0 (0.6)**	**Absolute positive**
**Teaching and learning strategies**		
(45) I am stimulated to actively participate in classroom	2.9 (0.7)	Could be improved
(46) The teaching strategy stimulates my thinking	2.9 (0.7)	Could be improved
(47) Teaching is student-centred (teaching addresses learning needs of individual students)	2.9 (0.7)	Could be improved
(48) Teaching is well integrated and focused	3.0 (0.6)	Absolute positive
**Physical classroom environment**		
(49) The teaching method develops my confidence	2.9 (0.8)	Could be improved
(50) The time for teaching is sufficient	3.0 (0.7)	Absolute positive
(51) My learning needs are addressed	2.9 (0.8)	Could be improved
(52) Teaching is focused on the teacher	2.5 (0.8)	Could be improved
(53) I can understand the lecturers in classrooms	3.0 (0.7)	Absolute positive
(54) I am able to meet the learning outcomes through the teaching and learning strategies used	3.0 (0.7)	Absolute positive
(55) Clinical training activities prepare the student to perform effectively in the clinical setting	3.2 (0.7)	Absolute positive
**Mean score**	**2.9 (0.5)**	**Could be improved**
**Nursing curriculum**		
(56) I am sure about the programme learning outcomes	3.0 (0.7)	Absolute positive
(57) The teaching and learning experience of the previous year prepared me well for this year	3.0 (0.9)	Absolute positive
(58) Time table arrangement allows for academic engagement	2.7 (0.9)	Could be improved
(59) Assessments are aligned to the outcomes provided in module guides	3.1 (0.7)	Absolute positive
(60) The curriculum provides an appropriate balance between theory and practice	2.9 (0.8)	Could be improved
(61) The learning outcomes are appropriate for the year level	3.1 (0.7)	Absolute positive
(62) The curriculum is organised in a way that facilitates my learning	3.0 (0.7)	Absolute positive
(63) The learning materials, including module guides, work books and so on, are clear	3.1 (0.7)	Absolute positive
(64) The programme thus far developed my ability to apply theory to practice	3.2 (0.6)	Absolute positive
(65) The programme thus far improved my problem-solving skills	3.1 (0.7)	Absolute positive
(66) The programme thus far developed my ability to think critically about the subject matter	3.2 (0.6)	Absolute positive
(67) The programme thus far helped me understand current issues in the nursing profession	3.2 (0.7)	Absolute positive
**Mean score**	**3.0 (0.5)**	**Absolute positive**

**Total mean score**	**195.2 (24.2)**	**Good**

SD, standard deviation.

## Discussion

The aim of the present study was to evaluate the educational environment as perceived by undergraduate nursing students at a SON. It also aimed to investigate whether the educational environment, or parts thereof, was perceived negatively or positively among undergraduate nursing students of different year level, gender or ethnicity.

### Perception of educational environment for the entire sample of undergraduate nursing students

Recent studies conducted across the world have been conclusive in finding that the majority of undergraduate nursing students perceive their educational environment as predominantly positive (Brown, Williams & Lynch 2011; Hamid, Faroukh & Mohammadhosein 2013; Imanipour et al. 2015; Victor, Ishtiaq & Parveen 2016). A more positive perception (high mean overall score) of the educational environment by nursing students indicates a more student-centred approach to teaching and learning (Roff 2005). The present study revealed similar results. The total mean score for the entire sample of undergraduate nursing students was 195.2 (72.8%) which was well between 135 and 201, indicating that generally the students’ perception of the educational environment was more positive than negative. In addition, the results were fairly consistent across the study subscales, ranging from 67.9% to 80%. However, these results fell short of achieving the ‘excellent’ ranking (total mean score of between 202 and 268). A conclusion that can be drawn from these results is that although the overall students’ perception is more positive and the identified educational environment is student-centred, the environment can nevertheless be further improved. The enhancement of the educational environment is likely to have a significant impact on the academic performance and retention of nursing students (Al Ayed & Sheik 2008; Arzuman et al. 2010; Till 2005). The findings presented in Table 5 provide an overview of subscales for potential interventions to improve the quality of the educational environment as perceived by the undergraduate nursing students.

### Perception of educational environment by year level

A positive perception of the educational environment was mutual for participants in all year levels of the undergraduate nursing programme. The total score per year level ranged from 186 to 202, indicating that the perception of the educational environment fell in the category of ‘good’. The subscale means scores ranging between 60.7% and 100% of the maximum scores also indicated a positive perception of the educational environment. These results are consistent with the findings of the majority of the studies conducted around the world (Brown et al. 2011; Hamid et al. 2013; Imanipour et al. 2015; Victor et al. 2016).

A few trends were noted between the year levels. Particularly, first-year BN students seemed to view their educational environment as more satisfactory than did second-, third- and fourth-year BN students with regard to both the on-campus and off-campus skills laboratories. Papathanasiou, Tsaras and Sarafis (2014) suggest that ‘students generally wish for a more positive clinical learning environment than what they have experienced, especially when it comes to issues related to satisfaction, individualisation and innovation’. Therefore, it is pivotal that skills laboratories where simulated clinical learning takes place are improved to ensure the development of critical thinking among nursing students (Henderson et al. 2010).

First- and second-year BN students viewed DR more favourably than did third- and fourth-year BN students. A comparative study conducted by He et al. (2012), comparing participants from two universities in the United States and China with the aim of ‘identifying the opinions of undergraduate students on the importance of internet-based information sources when they undertake academic tasks’, revealed that students use various DR including, but not limited to, search engines and social networking. Similarly, the findings of a cross-sectional study conducted by Johansson et al. (2014) in Sweden revealed that most nursing students regarded smart mobile devices to be useful in providing easy access to essential information to improve evidence-based practice, record keeping, planning their work and saving time. A conclusion that can be drawn is that improving access to efficient and reliable digital recourses will ensure a positive educational environment that promotes quality teaching and learning (He et al. 2012; Johansson et al. 2014; Thongmak 2013).

Likewise, the result of this study revealed that junior students (first- and second-year BN students) viewed NC more favourably than did the senior students (third- and fourth-year BN students). According to previous studies, students of an innovative curriculum tend to show more contentment with their educational environments compared with students of the traditional curriculum. The higher scores in the undergraduate nursing students’ perceptions towards their curriculum indicate a more student-centred curriculum (Aghamolaei & Fazel 2010; Wang, Zang & Shan 2009). Fourth-year BN students also seemed to rate the TLS implemented as less favourable. A mixed-method study conducted by Sinclair and Ferguson (2009) revealed that:

nursing students reported higher levels of satisfaction, effectiveness and consistency with their learning style when exposed to the combination of lecture and simulation than the control group, who were exposed to lecture as the only method of teaching and learning. (pp. 7–10)

Furthermore, a descriptive study conducted by Ozturk, Muslu and Dicle (2008) using the California Critical Thinking Disposition Inventory (CCTDI), which aimed at determining the critical thinking levels of undergraduate nursing students, revealed that nursing students who were exposed to problem-based learning (PBL) had higher critical thinking disposition scores as compared with their counterparts who were exposed to the traditional model. Therefore, it is vital that nurse educators integrate various teaching strategies that are problem based and encourage self-directed learning (Kong et al. 2014; Ozturk et al. 2008).

A general trend that emerged from the students’ perceptions of their educational environment by year level was that senior (third- and fourth-year) BN students viewed the educational environment at the selected university as less satisfactory than the junior (first- and second-year) BN students. The findings are consistent with those of Said, Rogayah and Hafizah (2009) and Hamid et al. (2013) who revealed reduced scores for senior students. Hamid et al. (2013) suggested that:

this trend could be due to the fact that students genuinely believed that their learning environment was deteriorating, and thus were psychologically tired of being a student and looking forward to leaving student life. (p. 61)

Conversely, contradictory findings noted in studies conducted by Till (2005) and Sayed and El-Sayed (2012) in Canada and the United Kingdom, respectively, revealed that third-year students had a more positive perception of their educational environment than the first- and second-year students. It is, however, important to acknowledge that first-year BN students had year-long modules, and therefore their assessments were yet to happen. Furthermore, it is also essential to note that service department teaching essentially occurs from second year. The same factors similarly apply to third- and fourth-year students by virtue of them having been studying longer. Junior students (first and second years) may be unable to give full account of the educational environment at the SON because of not having experienced the challenges that are faced by third- and fourth-year students at this stage of their training programme. This point may have implications for the interpretation of this result, and therefore it must be interpreted with caution.

### Perception of educational environment by ethnicity

Previous studies evaluating the perceptions of students of their educational environment categorised students based on their immigrant background (Avalos, Freeman & Dunne 2007; Palmgren & Chandratilake 2011). Some studies identified students of minority ethnic status to be at risk of experiencing difficulties in new educational environments (Maduwanthi, Mudalige & Atapattu 2015; Ostapczuk et al. 2012). These variations in the calcifications of ethnicity made it difficult to compare previous studies with the ethnic background of students as categorised in the present study. In this study, a positive perception of the educational environment was mutual for all ethnic groups. The total score per ethnic group ranged from 169 to 202, indicating that the perception of the educational environment across all racial groups fell in the category of ‘good’.

A few trends were noted between ethnic groups. Black students seemed to view their educational environment as more favourable than did coloured students and the category classified as ‘other’, particularly with regard to NC, both on-campus and off-campus skills laboratories, TLC and implemented TLS. These results provide evidence that it is imperative for the SON identified in this study to adopt a multicultural learning environment (Giddens 2008).

The present study revealed that ‘other’ category viewed the TLC less favourably than did white students. Interestingly, a study conducted by Avalos et al. (2007) in Ireland reported a statistically significant difference between Irish and non-Irish students’ perceptions of the TLC. In addition, a study conducted by Palmgren and Chandratilake (2011) in Sweden revealed similar findings between students of Swedish and non-Swedish ethnic background. These results should be interpreted with caution because of the vast contextual differences between the present study and previous studies. Furthermore, the sample size for the categories that were found to be statistically different was relatively small.

Taken together, it can be concluded that black students viewed their educational environment as more favourable than did other ethnic groups. This finding could be explained by the fact that the majority of black students at the selected university are predominantly from previously disadvantaged educational backgrounds and consequently might be more appreciative of anything that was better than what they had previously experienced (University of the Western Cape 2018).

### Perception of educational environment by gender

Previous studies conducted in the medical field comparing gender differences revealed that female students were more positive about their educational environment compared to their male counterparts (Jawaid et al. 2013; Lokuhetty et al. 2010; Nahar et al. 2010; Riquelme et al. 2009). However, the same cannot be said of the nursing field. Similar to the study conducted by Victor et al. (2016) in Pakistan, the results of the present study revealed that male students viewed the educational environment more favourably than did their female counterparts. However, it must be acknowledged that the trend was not statistically significant for all subscales of the educational environment (off-campus SL, UL, DR, TLC and TLS) at the identified SON. These findings may result from the fact that male nursing students are a minority group and are known to receive special treatment from educators as well as clinical supervisors, and therefore they might have a preponderance of positive experiences (Moss-Racusin et al. 2012). Kouta and Kaite (2011) indicated that gender bias in nursing education could have an influence on perceptions of the educational environment.

## Implications for nursing education

Although it is acknowledged that academic performance and success is unquestionably a complex phenomenon with various contributing factors (Jeffreys 2015; Mthimunye, Daniels & Pedro 2018), nursing schools need to take steps to ensure that the educational environment in which they expect their students to thrive promotes a quality learning process. The findings of the present study are vital in terms of understanding the environmental needs of undergraduate nursing students in a South African educational context. The implications for nursing education emerging from this study include the necessity of improving the following:

- conditions of the PCE: this includes creating a pleasant place to work with adequate ventilation, temperature regulation and adequate seating arrangements
- conditions of the SL environment: this includes ensuring adequate ventilation, temperature regulation, accessibility and ensuring appropriate and sufficient equipment necessary for practice of required clinical skills
- DR as well as making provision for Internet access for students who reside off-campus
- TLS adopted at the identified SON.

## Limitations and recommendations

Although this study provides crucial evidence regarding the educational environment at the SON, it would be invaluable to conduct a similar study that includes students from other departments in the community and health science faculty. The limitation that should be acknowledged in this study is that because of financial reasons and time constraints an adjusted sample size of 287 participants was calculated to ensure a sample that is representative of the study population (Dean et al. 2013). Similar studies should be conducted with larger samples at other universities and nursing schools in South Africa and around the world to increase the generalisability of the findings beyond the investigated university. For future studies, we recommend a qualitative follow-up study with the participants to gain in-depth understanding of the aspects that need to be improved. In addition, it would be interesting for future studies to evaluate the relationship between students’ perceptions of their educational environment and academic performance.

## Conclusion

This study’s findings conclude that the selected participants at the identified university generally perceived their educational environment as being more positive than negative. Regarding students’ general perceptions of the subscales, enhancements are required in the PCE, skills laboratories (both on-campus and off-campus), DR and the implemented TLS. In contrast, and completing the range of subscales, the students’ perceptions of the subscales UL, TLC and NC seem to require minimal enhancements, if any. It is essential for university management and the SON to prioritise the suggested improvements based on the results of this study to create an educational environment that promotes quality learning.

## Footnotes

1 Statistics South Africa continues to classify people into population groups as moving away from the pre-1994 apartheid-based system. This classification uses a population group-based classification system that is no longer based on a legal definition, but rather on self-classification (Statistics South Africa 2016).

## References

[CIT0001] Aghamolaei T. & Fazel I., 2010, ‘Medical students’ perceptions of the educational environment at an Iranian Medical Sciences University’, *BMC Medical Education* 10(1), 87. 10.1186/1472-6920-10-87 21114818 PMC3001739

[CIT0002] Aiken L.H., 2011, ‘Nurses for the future’, *New England Journal of Medicine* 364(3), 196–198. 10.1056/NEJMp1011639 21158647

[CIT0003] Al Ayed I.H. & Sheik S.A., 2008, ‘Assessment of the educational environment at the College of Medicine of King Saud University, Riyadh’, *Eastern Mediterranean Health Journal* 14(4), 953–959. 19166179

[CIT0004] Arzuman H., Yusoff M.S. & Chit S.P., 2010, ‘Big Sib students’ perceptions of the educational environment at the School of Medical Sciences, Universiti Sains Malaysia, using Dundee Ready Educational Environment Measure (DREEM) inventory’, *Malaysian Journal of Medical Sciences* 17(3), 40–47. PMC3216174 22135548

[CIT0005] Avalos G., Freeman C. & Dunne F., 2007, ‘Determining the quality of the medical educational environment at an Irish medical school using the DREEM inventory’, *Irish Medical Journal* 100(7), 522–525. 17886524

[CIT0006] Baeten M., Kyndt E., Struyven K. & Dochy F., 2010, ‘Using student-centred learning environments to stimulate deep approaches to learning: Factors encouraging or discouraging their effectiveness’, *Educational Research Review* 5(3), 243–260. 10.1016/j.edurev.2010.06.001

[CIT0007] Benner P., 2012, ‘Educating nurses: A call for radical transformation – How far have we come?’, *Journal of Nursing Education* 51(4), 183–184. 10.3928/01484834-20120402-01 22476535

[CIT0008] Billings D.M. & Halstead J.A., 2015, *Teaching in nursing-e-book: A guide for faculty*, Elsevier Health Sciences, St.Louis, MO.

[CIT0009] Brown T., Williams B. & Lynch M., 2011, ‘The Australian DREEM: Evaluating student perceptions of academic learning environments within eight health science courses’, *International Journal of Medical Education* 2, 94. 10.5116/ijme.4e66.1b37

[CIT0010] Bruce J.C., Klopper H. & Mellish J., 2011, *Teaching and learning the practice of nursing*, Heinemann, Portsmouth, NH.

[CIT0011] Cheon J., Lee S., Crooks S.M. & Song J., 2012, ‘An investigation of mobile learning readiness in higher education based on the theory of planned behavior’, *Computers & Education* 59(3), 1054–1064. 10.1016/j.compedu.2012.04.015

[CIT0012] Cleveland B. & Fisher K., 2014, ‘The evaluation of physical learning environments: A critical review of the literature’, *Learning Environments Research* 17(1), 1–28. 10.1007/s10984-013-9149-3

[CIT0013] Colbert-Getz J.M., Kim S., Goode V.H., Shochet R.B. & Wright S.M., 2014, ‘Assessing medical students’ and residents’ perceptions of the learning environment: Exploring validity evidence for the interpretation of scores from existing tools’, *Academic Medicine: Journal of the Association of American Medical Colleges* 89(12), 1687–1693. 10.1097/ACM.0000000000000433 25054415

[CIT0014] Cornell S. & Hartmann D., 2007, *Ethnicity and race: Making identities in a changing world*, Pine Forge Press, Thousand Oaks, CA.

[CIT0015] Davies D., Jindal-Snape D., Collier C., Digby R., Hay P. & Howe A., 2013, ‘Creative learning environments in education – A systematic literature review’, *Thinking Skills and Creativity* 8, 80–91. 10.1016/j.tsc.2012.07.004

[CIT0016] Davis A.H. & Kimble L.P., 2011, ‘The essentials of baccalaureate education for professional nursing practice’, *Journal of Nursing Education* 50(11), 605. 10.3928/01484834-20110715-01 21751764

[CIT0017] Dean A.G., Sullivan K.M. & Soe M.M., 2013, *OpenEpi: Open source epidemiologic statistics for public health**,* viewed June 2017, from http://www.openepi.com/SampleSize/SSCC.htm

[CIT0018] Denscombe M., 2014, *The good research guide: For small-scale social research projects*, McGraw-Hill Education, London.

[CIT0019] Denz-Penhey H. & Murdoch J.C., 2009, ‘A comparison between findings from the DREEM questionnaire and that from qualitative interviews’, *Medical Teacher* 31(10), e449–e453. 10.3109/01421590902849552 19877851

[CIT0020] Field A., 2013, *Discovering statistics using IBM SPSS statistics*, Sage, Thousand Oaks, CA.

[CIT0021] Giddens J.F., 2008, ‘Achieving diversity in nursing through multicontextual learning environments’, *Nursing Outlook* 56(2), 78–83. 10.1016/j.outlook.2007.11.003 18374802

[CIT0022] Hamid B., Faroukh A. & Mohammadhosein B., 2013, ‘Nursing students’ perceptions of their educational environment based on the DREEM model in an Iranian University’, *The Malaysian Journal of Medical Sciences* 20(4), 56–63. PMC3773353 24043997

[CIT0023] He D., Wu D., Yue Z., Fu A. & Thien Vo K., 2012, ‘Undergraduate students’ interaction with online information resources in their academic tasks: A comparative study’, *Aslib Proceedings* 64(6), 615–640, Emerald Group Publishing Limited. 10.1108/00012531211281715

[CIT0024] Henderson A., Creedy D., Boorman R., Cooke M. & Walker R., 2010, ‘Development and psychometric testing of the clinical learning organisational culture survey (CLOCS)’, *Nurse Education Today* 30(7), 598–602. 10.1016/j.nedt.2009.12.006 20064678

[CIT0025] Imanipour M., Sadooghiasl A., Ghiyasvandian S. & Haghani H., 2015, ‘Evaluating the educational environment of a nursing school by using the DREEM inventory’, *Global Journal of Health Science* 7(4), 211–216. 10.5539/gjhs.v7n4p211 25946923 PMC4802054

[CIT0026] Jawaid M., Raheel S., Ahmed F. & Aijaz H., 2013, ‘Students’ perception of educational environment at Public Sector Medical University of Pakistan’. *Journal of Research in Medical Sciences: The Official Journal of Isfahan University of Medical Sciences* 18(5), 417–421. PMC3810578 24174949

[CIT0027] Jeffreys M.R., 2015, ‘Jeffrey’s nursing universal retention and success model: Overview and action ideas for optimizing outcomes A–Z’, *Nurse Education Today* 35(3), 425–431. 10.1016/j.nedt.2014.11.004 25434347

[CIT0028] Johansson P., Petersson G., Saveman B.I. & Nilsson G., 2014, ‘Using advanced mobile devices in nursing practice–the views of nurses and nursing students’, *Health Informatics Journal* 20(3), 220–231. 10.1177/1460458213491512 25183609

[CIT0029] Kong L.N., Qin B., Zhou Y.Q., Mou S.Y. & Gao H.M., 2014, ‘The effectiveness of problem-based learning on development of nursing students’ critical thinking: A systematic review and meta-analysis’, *International Journal of Nursing Studies* 51(3), 458–469. 10.1016/j.ijnurstu.2013.06.009 23850065

[CIT0030] Korucu A.T. & Alkan A., 2011, ‘Differences between m-learning (mobile learning) and e-learning, basic terminology and usage of m-learning in education’, *Procedia-Social and Behavioral Sciences* 15, 1925–1930. 10.1016/j.sbspro.2011.04.029

[CIT0031] Kouta C. & Kaite C.P., 2011, ‘Gender discrimination and nursing: A literature review’, *Journal of Professional Nursing* 27(1), 59–63. 10.1016/j.profnurs.2010.10.006 21272837

[CIT0032] Lokuhetty M.D., Warnakulasuriya S.P., Perera R.I., De Silva H.T. & Wijesinghe H.D., 2010, ‘Students’ perception of the educational environment in a medical faculty with an innovative curriculum in Sri Lanka’, *South-East Asian Journal of Medical Education* 9(4), 9–16.

[CIT0033] Macnee C.L. & McCabe S., 2008, *Understanding nursing research: Using research in evidence-based practice*, Lippincott Williams & Wilkins, Philadelphia, PA.

[CIT0034] Maduwanthi W.T.S., Mudalige S.K.K. & Atapattu N.S.B.M., 2015, ‘Academic performance and the perception about the educational environment: A comparison between ethnic minority and majority students following three degree programs’, *Tropical Agricultural Research and Extension* 18(3), 107–111. 10.4038/tare.v18i3.5333

[CIT0035] Miles S., Swift L. & Leinster S.J., 2012, ‘The Dundee Ready Education Environment Measure (DREEM): A review of its adoption and use’, *Medical Teacher* 34(9), e620–e634. 10.3109/0142159X.2012.668625 22471916

[CIT0036] Moss-Racusin C.A., Dovidio J.F., Brescoll V.L., Graham M.J. & Handelsman J., 2012, ‘Science faculty’s subtle gender biases favor male students’, *Proceedings of the National Academy of Sciences of the United States of America* 109(41), 16474–16479. 10.1073/pnas.1211286109 22988126 PMC3478626

[CIT0037] Moultrie T.A. & Timæus I.M., 2003, ‘The South African fertility decline: Evidence from two censuses and a Demographic and Health Survey’, *Population Studies* 57(3), 265–283. 10.1080/0032472032000137808 14602529

[CIT0038] Mthimunye K.D.T., Daniels F.M. & Pedro A., 2018, ‘Predictors of academic performance among second-year nursing students at a university in the Western Cape’, *South African Journal of Higher Education* 32(1), 192–215.

[CIT0039] Nahar N., Talukder M.H.K., Khan M.T.H., Mohammad S. & Nargis T., 2010, ‘Students’ perception of educational environment of medical colleges in Bangladesh’, *Bangabandhu Sheikh Mujib Medical University Journal* 3(2), 97–102.

[CIT0040] Ostapczuk M., Hugger A., De Bruin J., Ritz-Timme S. & Rotthoff T., 2012, ‘DREEM on, dentists! students’ perceptions of the educational environment in a German dental school as measured by the Dundee Ready Education Environment Measure’, *European Journal of Dental Education* 16(2), 67–77. 10.1111/j.1600-0579.2011.00720.x 22494304

[CIT0041] Ozturk C., Muslu G.K. & Dicle A., 2008, ‘A comparison of problem-based and traditional education on nursing students’ critical thinking dispositions’, *Nurse Education Today* 28(5), 627–632. 10.1016/j.nedt.2007.10.001 18054412

[CIT0042] Palmgren P.J. & Chandratilake M., 2011, ‘Perception of educational environment among undergraduate students in a chiropractic training institution’, *Journal of Chiropractic Education* 25(2), 151–163. 10.7899/1042-5055-25.2.151 22069340 PMC3204951

[CIT0043] Papathanasiou I.V., Tsaras K. & Sarafis P., 2014, ‘Views and perceptions of nursing students on their clinical learning environment: Teaching and learning’, *Nurse Education Today* 34(1), 57–60. 10.1016/j.nedt.2013.02.007 23481172

[CIT0044] Perneger T.V., Courvoisier D.S., Hudelson P.M. & Gayet-Ageron A., 2015, ‘Sample size for pre-tests of questionnaires’, *Quality of Life Research* 24(1), 147–151. 10.1007/s11136-014-0752-2 25008261

[CIT0045] Polit D.F. & Beck C.T., 2010, *Essentials of nursing research: Appraising evidence for nursing practice*, Lippincott Williams & Wilkins, Philadelphia, PA.

[CIT0046] Rahman N.I., Aziz A.A., Zulkifli Z., Haj M.A., Mohd Nasir F.H., Pergalathan S. et al., 2015, ‘Perceptions of students in different phases of medical education of the educational environment: Universiti Sultan Zainal Abidin’, *Advances in Medical Education and Practice* 6, 211–222. 25848333 10.2147/AMEP.S78838 PMC4378299

[CIT0047] Riquelme A., Oporto M., Oporto J., Mendez J.I., Viviani P., Salech F. et al., 2009, ‘Measuring students’ perceptions of the educational climate of the new curriculum at the Pontificia Universidad Catolica de Chile: Performance of the Spanish translation of the Dundee Ready Education Environment Measure (DREEM)’, *Education for Health* 22(1), 112. 19953435

[CIT0048] Roff S., 2005, ‘The Dundee Ready Educational Environment Measure (DREEM) – A generic instrument for measuring students’ perceptions of undergraduate health professions curricula’, *Medical Teacher* 27(4), 322–325. 10.1080/01421590500151054 16024414

[CIT0049] Said N.M., Rogayah J. & Hafizah A., 2009, ‘A study of learning environments in the Kulliyyah (Faculty) of Nursing, International Islamic University Malaysia’, *The Malaysian Journal of Medical Sciences* 16(4), 15. PMC3216132 22135508

[CIT0050] Statistics South Africa, 2016, *Community Survey 2016*. Statistical release P0301, viewed n.d., from http://cs2016.statssa.gov.za/wp-content/uploads/2016/07/NT-30-06-2016-RELEASE-for-CS-2016-_Statistical-releas_1-July-2016.pdf

[CIT0051] Sayed H. & El-Sayed N., 2012, ‘Student’s perceptions of the educational environment of the nursing program in Faculty of Applied medical Sciences at Umm-Al-Qura University, KSA’, *Journal of American Science* 8(14), 69–75.

[CIT0052] Sinclair B. & Ferguson K., 2009, ‘Integrating simulated teaching/learning strategies in undergraduate nursing education’, *International Journal of Nursing Education Scholarship* 6(1), 1–11. 10.2202/1548-923X.1676 19341357

[CIT0053] Tavakol M. & Dennick R., 2011, ‘Making sense of Cronbach’s alpha’, *International Journal of Medical Education* 2, 53. 10.5116/ijme.4dfb.8dfd 28029643 PMC4205511

[CIT0054] Thongmak M., 2013, ‘Social network system in classroom: Antecedents of edmodo© adoption’, *Journal of e-Learning and Higher Education* 2013(1), 1–15.

[CIT0055] Till H., 2005, ‘Climate studies: Can students’ perceptions of the ideal educational environment be of use for institutional planning and resource utilization?’, *Medical Teacher* 27(4), 332–337. 10.1080/01421590400029723 16024416

[CIT0056] University of the Western Cape, 2018, About UWC, viewed 25 June 2018, from https://www.uwc.ac.za/Pages/History.aspx

[CIT0057] Veerapen K. & McAleer S., 2010, ‘Students’ perception of the learning environment in a distributed medical programme’, *Medical Education Online* 15(1), 5168. 10.3402/meo.v15i0.5168 PMC2946853 20922033

[CIT0058] Victor G., Ishtiaq M. & Parveen S., 2016, ‘Nursing students’ perceptions of their educational environment in the bachelor’s programs of the Shifa College of Nursing, Pakistan’, *Journal of Educational Evaluation for Health Professions* 13, 43. 10.3352/jeehp.2016.13.43 28013314 PMC5286213

[CIT0059] Wang J., Zang S. & Shan T., 2009, ‘Dundee ready education environment measure: Psychometric testing with Chinese nursing students’, *Journal of Advanced Nursing* 65(12), 2701–2709. 10.1111/j.1365-2648.2009.05154.x 19941551

[CIT0060] World Health Organization, 2013, *Transforming and scaling up health professionals’ education and training: World Health Organization guidelines 2013*, World Health Organization, Geneva. 26042324

[CIT0061] Yusoff M.S.B., 2012, ‘The Dundee ready educational environment measure: A confirmatory factor analysis in a sample of Malaysian medical students’, *International Journal of Humanities and Social Science* 2(16), 313–321.

